# Insight in Psychotic vs. Non-Psychotic Disorders: Understanding Mental States and Their Impact on Behavior and Decision-Making

**DOI:** 10.1192/j.eurpsy.2026.11799

**Published:** 2026-07-31

**Authors:** A. Halaj

**Affiliations:** University of Haifa, Haifa, Israel

## Abstract

**Introduction:**

Insight—the awareness and understanding of one’s own mental state—is a crucial element of mental health, shaping how individuals interpret their experiences, engage with the world, and make decisions about behaviors, both adaptive and maladaptive. It extends beyond the recognition of symptoms to include the capacity to understand how thoughts, emotions, and perceptions influence behavior and self-awareness. This reflective capacity is essential for clinical assessment, treatment engagement, and effective decision-making.

**Objectives:**

To examine how insight manifests differently in psychotic versus non-psychotic disorders and to explore its intersection with metacognition, decision-making, and behavior.

**Methods:**

The presentation integrates conceptual, clinical, and ethical perspectives to compare patterns of insight across psychotic and non-psychotic conditions. It draws on existing research regarding assessment approaches and therapeutic strategies that address these differences.

**Results:**

In psychotic disorders such as schizophrenia, profound disruptions in reality testing, hallucinations, and delusions lead to marked impairments in awareness and metacognition, strongly influencing treatment outcomes. In non-psychotic disorders, including mood and anxiety disorders, insight difficulties are more subtle and complex. Individuals may recognize their symptoms but often struggle to connect internal experiences with external behaviors. These nuanced deficits are harder to detect and complicate treatment planning and engagement. Enhancing insight may support therapeutic progress but can also increase distress.

**Conclusions:**

Expanding the study of insight beyond psychosis enriches the understanding of non-psychotic disorders and contributes to more precise, patient-centered treatment approaches. Considering both clinical and ethical dimensions is essential for improving assessment and tailoring interventions across the spectrum of mental health conditions.

**Disclosure of Interest:**

None Declared

